# *SLC1A3* C3590T but not *BDNF* G196A is a predisposition factor for stress as well as depression, in an adolescent eastern Indian population

**DOI:** 10.1186/s12881-020-0993-6

**Published:** 2020-03-14

**Authors:** Madhumita Ghosh, Akhtar Ali, Shobhna Joshi, Adya Shankar Srivastava, Madhu G. Tapadia

**Affiliations:** 1 Cytogenetics Laboratory, Department of Zoology, Banaras Hindu University, Varanasi, 221005 India; 2 Centre for Genetic Disorders, Faculty of Science, Banaras Hindu University, Varanasi, 221005 India; 3 Department of Psychology, Faculty of Arts, Banaras Hindu University, Varanasi, 221005 India; 4 Department of Psychiatry, Institute of Medical Sciences, Banaras Hindu University, Varanasi, 221005 India

**Keywords:** Depression, Stress, Adolescent, *SLC1A3*, *BDNF*, Polymorphism, India

## Abstract

**Background:**

Adolescence is a distinctive stage of various changes and is noted as peak age for onset of many psychiatric disorders, especially linked to stress and depression. Several genetic variations are being increasingly known to be linked with stress and depression. The polymorphisms in two such genes, the *BDNF* and *SLC1A3*, have been reported to be linked with either depression/stress or with suicidal behaviour. These genes have not been validated in Indian population, and therefore there is a need to investigate these genes in Indian population. The present study was undertaken to test whether the known polymorphisms *SLC1A3* C3590T, *SLC1A3* C869G and *BDNF* G196A are associated or not with stress or depression in an eastern Indian population.

**Methods:**

A case-control association study was performed with 108 cases having variable levels of stress and depression and 205 matched controls. Detection of stress and depression was done by using standard instruments as PSS and CES-D, respectively and demographic profile was obtained for each individual on the basis of personal data sheet. Genotyping for the selected polymorphisms was performed by PCR followed by restriction digestion.

**Results:**

The SNP *SLC1A3* C3590T was found to be associated with stress and depression (*p* = 0.0042, OR = 2.072). Therefore, the T allele increases the risk by more than two folds for stress and depression in the present population. The other allele of *SLC1A3*, G869C, as well as *BDNF* G196A were not associated with stress or depression in the population studied.

**Conclusion:**

*SLC1A3* C3590T is a predisposition factor for stress and depression in an eastern Indian population, whereas *SLC1A3* G869C and *BDNF* G196A were not found to be a risk factor. Therefore, presence of T allele of *SLC1A3* C3590T, may predict the development of stress and depression in an individual. This may also help in the understanding of pathophysiology of the disease. However, these findings warrant a wider study in Indian populations and would be of significance in understanding the predisposition of stress and depression in this population.

## Introduction

Stress is considered as a non-specific response of the body to any demand for change [1] or in medical terms any bodily change which causes mental tension; while depression is a mood disorder characterized by low mood, sadness and general loss of interest in life [2]. Adolescence or teenagers (13–21 years of age) is the stage of remarkable physical and behavioural changes in the brain and body and is noted as the peak age of onset for many psychiatric disorders, particularly linked with stress and depression [3]. Nevertheless, most of the teenagers successfully steer this transition stage and become a self-sufficient member of the society. More than one genetic variant(s) in conjunction with environmental factors are likely to increase risk for stress and depression.

Twin studies in past have implicated that heritability of depression ranges from 30 to 40% indicating a significant level of genetic involvement in the development of stress and depression [4–6]. Several genes and gene families have been scanned for their association with depression and one of them is the SLC1 gene family of solute carriers [7] which encodes the excitatory amino acid transporters (EAAT). In humans, five isoforms of EAATs (EAAT1–5) are known which have different tissue localization. EAAT 1 (coded by *SLC1A3* located on chromosome no. 5p13.2) transports glutamate in the brain. Glutamate is a ubiquitous amino acid, which acts as the major excitatory neurotransmitter in numerous projection fibres from cortical and subcortical structures [8], and also plays a crucial role in maintaining the activity of other neurotransmitter systems [9]. It is also recognized that the excitatory properties of glutamate remain under a delicate balance; otherwise it exerts damage or excitotoxicity that can range from cellular malfunction to extensive tissue injury [9]. In brain, specific high affinity glutamate transporters remove glutamate, which ends neurotransmitter activity until the next release of neurotransmitters occur [9]. Disturbance in glutamate uptake from synapses have been linked to reduced sensitivity to feel good, a symptom of depression [10]. Similarly, glutamate receptor AMPA subunit-1 knockout mice showed higher helplessness, lower serotonin and nor-epinephrine levels, disturbed glutamate homeostasis with high glutamate levels and increase in NMDA receptor expression. In ischemia, epilepsy, traumatic brain injury, acute or chronic excitotoxicity associated with the excessive glutamate release is known to be the main cause of tissue damage. GWAS studies conducted for depression in last several years have identified different genetic loci [11], like tSNARE1 [12], and MALDIL1 [13] and several molecular pathways involved in cell adhesion and membrane scaffolding [14]. Microarray analysis has shown considerable reduction in the expression of the members of the SLC1 family of membrane transporters - SLC1A2 and SLC1A3, but increase levels of SLC17A7, in the depression cases compared to controls [15] suggesting their association with occurrence and/or severity of depression. SNP in *SLC1A3* C3590T (rs 2269272) is a polymorphism in 3′ UTR, has shown association with suicidal behaviour [16]. The other SNP, *SLC1A3* C869G (rs137852619), a missense polymorphism in protein coding region leading to conversion from C to G at 869 position, changes proline to arginine and found to be pathogenic, recorded in database but not validated with larger samples. The molecular functioning, genetic interactions and expression profiling of *SLC1A3* gene was also studied, but a plausible relationship between sequence variations in *SLC1A3* gene and depression, have not been studied so far.

A second important candidate gene is *BDNF* (brain-derived neurotrophic factor; located on chromosome 11p13) whose molecular pathology has been extensively studied in depression cases [17]. *BDNF* gene codes for a neurotrophin, which is highly expressed in the central nervous system, particularly in the hippocampus region. It plays elementary role in proliferation, differentiation and survival of neuronal cells, as well as regulating synaptic plasticity and connectivity in the adult brain (memory acquisition and consolidation). The majority of BDNF is secreted via the regulated pathway and is derived from both pre- and postsynaptic sites. A widely accepted “*neurotrophic hypothesis*”, postulates that a reduction in *BDNF* expression is involved in the pathophysiology of major depression [18], while anti-depressant increases the expression of *BDNF* in hippocampus [19]. Further, prenatal stress has been shown to alter BDNF expression and signalling [20].

A single nucleotide polymorphism, G196A (rs6265), results in an amino-acid substitution from valine to methionine at codon 66 (Val66Met) in the prodomain of *BDNF* [21, 22]. Met allele carriers have shown to exhibit increased harm avoidance and also shows a higher prevalence of depression. A recent review proposed BDNF stress-sensitivity hypothesis which states that disruption of endogenous BDNF activity potentiates sensitivity to stress [23]. Val66Met polymorphism is common in human population; however the allele frequencies have been shown to be varying in specific ethnic backgrounds [24, 25]. A considerably higher frequency of Met allele in poor episodic memory performance, a symptom often observed in subjects with depression [26] and also in stress related psychiatric disorders have been reported [23]. Subsequently, in-vitro analysis in animal models have suggested that ‘Met’ allele alters the sorting and secretion of proBDNF, such that less regulated (activity dependent) secretion is likely to occur in carriers of at least one ‘Met’ allele [27]. In spite of the ample evidences, inconsistencies persists [28, 29], in conceding the role of *BDNF* G196A polymorphism with mood disorders, which might also be associated with the lack of statistical power in studies of small sample size, as well as variations across studies in inclusion criteria, such as the definition of psychiatric phenotype [24, 30]. Further, role of sex hormones and various other genetic, hormonal and environmental factors have been implicated in modulating BDNF function [25, 29, 31–35]. Despite a higher risk of depression in Asian and Indian populations [25, 36], extensive studies targeting polymorphisms in *BDNF* have never been executed in these populations so far.

With the aim to test the hypothesis whether the known polymorphism in two candidate genes:

*SLC1A3* (C3590T and C869G) and *BDNF* (G196A) are associated with stress and depression in adolescent population of eastern Uttar Pradesh (India), a control-case association study has been undertaken.

## Methods and materials

### Participants

In the present study, a total of 313 adolescent participants aged 16 to19 years were recruited, out of which 205 were controls with no stress and depression and 108 were the cases having variable degrees of both stress and depression. The recruitment of cases were majorly from Sir Sundarlal Hospital, Banaras Hindu University, a few from Deva Clinic, Kamachha, Varanasi and a few schools chosen for this study. All the participants belong to Eastern Uttar Pradesh, India. Prior to conducting this study approval from ethical committee of Institute of Science, Banaras Hindu University, Varanasi, was taken. A written informed consent from each participant was obtained prior to conduction of the study. A clinical examination was conducted for all participants by a psychiatrist and followed a questionnaire developed from “Percieved stress scale” (PSS) [37] and “Center for epidemiologic Studies Depression” (CES-D) [38], which had major components including: (a) general information; (b) the chief complain (presenting problem(s) and the context in which they occur); c) past history (medical, personal, family histories and premorbid behaviour) d) mental status examination, e) diagnosis according to *DSM-5* guidelines [39] and lastly f) treatment plan for the diagnosis of stress and depression.

### Diagnosis of stress and depression

Detection and diagnosis of stress and depression was done according to DSM-V criteria [39] and screening questionnaires, as well as few semi structured questionnaires were administered on adolescents both for stress (PSS) and depression (CES-D).

*Stress:* The following criteria were used for diagnosis of stress: less/no control over irritation, anger on trivial things, unexpected circumstances makes individual upset, coping failure at work/ home or both, work overload, feeling of being subordinate, feeling nervous /stressed. The scores obtained on these points were computed using standard protocol.

*Depression:* Clinical depression was diagnosed by a psychiatrist which had the following criteria: Loss/ no interest in work, activity level decreased, increased thoughts of guilt/ worthlessness, disturbed appetite (reduced or increased), sleep duration (reduced or increased), energy levels of body (reduced or increased); impaired/ low concentration, irritable mood and suicidal thoughts or attempts of suicide. The severity level on these parameters was used to calculate the disease-score for each of the participants.

### Isolation of DNA from saliva

Saliva samples of participating adolescents were collected in sterilized vials in 1X phosphate buffered saline and then kept in − 70 °C till use. The samples were then thawed, vortexed and pelleted down by centrifugation at 6000 rpm for 5 min at 4 °C, cells were digested by SDS proteinase K treatment at 65 °C and DNA isolation was done following phenol chloroform extraction method as per standard protocol and precipitation by ethanol. DNA was air dried at 37 °C, dissolved in 25 μl of 1XTris-EDTA (pH =8.0) and was stored at 10 °C. Spectrophotometer (Nanodrop, Thermofisher®, USA) was used to check the quality and quantity of DNA.

### Genotyping through polymerase chain reaction (PCR) followed by restriction digestion

Primer 3 (v.0.4.0) (http://bioinfo.ut.ee/primer3-0.4.0/primer3/) and Primer-BLAST (https://www.ncbi.nlm.nih.gov/tools/primer-blast/) tools were used to design region specific primers. In PCR, denaturation was done at 95 °C, primers were annealed at temperatures accordingly (see Table 1) and elongation at 72 °C for 30 s, this cycle was repeated for 35 cycles. Final extension was carried out at 72 °C for 3 to 5 min as required, in thermocycler (ABI, Veriti, ABI®, CA, USA). Negative template control was taken without DNA for ensuring no contamination. The PCR products were checked on 2% agarose gel to ensure correct amplicon size. Desired endonucleases as *Nla*III (NEB®, CA, USA) for *BDNF* G196A, *Pfl*F1(NEB®, CA, USA) for *SLC1A3* C3590T and *Bst*U1(NEB®, CA, USA) for *SLC1A3* G869C were used for digestion of PCR products for genotyping as indicated in Table 2. After digestion the reaction was stopped by adding1μl 0.5 N EDTA and finally the digested products were checked on 4% agarose gel by electrophoresis.

**Table 1:** Primers used for detecting SNPs in *BDNF* and *SLC1A3* gene with optimized temperatures and product size

Gene	Primers		Annealing Temp	Product size
	Forward	Reverse		
*BDNF*	5’AAAGAAGCAAACATCCGAGGACAA3’	5’ATTCCTCCAGCAGAAAGAGAAGAGG3’	61 °C	274
*SLC1A3* C3590T (3’UTR)	5’TGGAGTGGGGTTTGCTTTTG3’	5’GCCAGAAACTCAAACACAGC3’	60 °C	232
*SLC1A3* G869C (Coding region)	5’ATTTCTGTGGACTAGCTTGGTG3’	5’TTACCAAGAAGTAGAGGAGTGGC3’	63 °C	223

**Table 2:** Amplicon size, restriction endonucleases used and allele wise expected size of fragments after restriction digestion for the three SNPs studied

SNP	Expected amplicon size (bp)	Restriction endonuclease used	Allele-wise expected fragment size (bp)
*BDNF* G196A (rs 6265)	274	*Nla*III (5′.... CATG^....3′) (5′...^GTAC......3’	GG = 216, 58 GA = 216, 139, 77, 58 AA = 139, 77, 58
*SLC1A3* C3590T (rs 2269272)	232	*Pfl*F1 (5′..GACN^NNGTC..3′) (3′..CTGNN^NCAG..5′)	CC = 157, 75 CT = 232, 157, 75 TT = 232
*SLC1A3* G869C (rs 137852619)	223	*Bst*U1 (5′..CG^CG..3′) (3′..GC^GC..5′)	GG = 223 GC = 223, 152, 71 CC = 152, 71

^ represent the recognition site

### Statistical analysis

Analysis of genotype frequencies and allelic frequencies of two polymorphisms of *SLC1A3* C3590T and *SLC1A3* G869C and single polymorphism of *BDNF* G196A, in cases and controls were done separately, to test whether the genotypes are in Hardy-Weinberg equilibrium or not (*p* value > 0.05). For statistical significance between genotypic and allelic distribution, Chi-square test and correlation matrix, were calculated between various factors. The analysis was done in the form of frequency tables, contingency table analysis using SPSS version 20 (IBM®, Armonk, NY, USA). Odds ratio was calculated to describe the (approximate) increase in risk associated with the non-reference genotype compared to the reference genotype and 95% confidence interval (CI) was computed using GraphPadInStat version 3.0. Throughout analysis, *p* value of < 0.05 was taken to be statistically significant.

## Results

### Association of SLC1A3 C3590T (rs 2269272) polymorphism with stress and depression

**Fig. 1:**
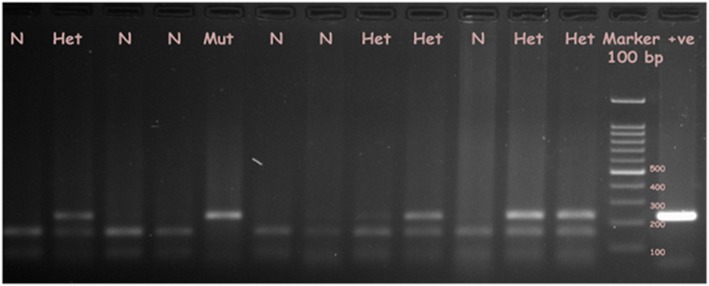
Restriction digestion by *Pfl*F1 of *SLC1A3* C3590T amplicon of representative individuals. The amplicon of *SLC1A3* C3590T after digestion with the restriction endonuclese, *Pfl*F1, in normal condition (i.e when C is present at 3590 position) shows two fragments of DNA, 157 and 75 bp (lane 1, 3, 4, 6, 7, 10). A substitution of T in place of C, causes loss of restriction site and only one band of 232 bp (full sized amplicon; lane 5) is obtained. Heterozygotes on the other hand shows three bands of 232, 157 and 75 bp (lane 2, 8, 9, 11, 12)

The amplicon of *SLC1A3* covering C3590T after digestion with the restriction endonuclease, *Pfl*F1, produce two fragments of DNA of 157 bp and 75 bp. But a substitution of T in place of C, causes loss of restriction site and thus single band of (full sized amplicon) 232 bp is obtained. Heterozygotes, on the other hand, shows three bands of 232, 157 and 75 bp (Fig. 1). Table 3 summarizes genotypic distribution of *SLC1A3* C3590T in both cases and controls. Statistically significant difference was observed between cases and controls where the frequencies of CC, CT, TT were 46.3, 50.9 and 2.8%, respectively, for cases and 63.4, 33.7 and 2.9%, respectively, for controls (*p* = 0.0115, χ^2^ = 8.934). The genotypic distribution in different groups according to three different models of association were analyzed, out of which significant differences in genotypic distributions was exhibited by dominant (CT + TT vs. CC) (*p* = 0.0052, χ^2^ = 7.796) and co-dominant models (CT vs. CC) (*p* = 0.0042, χ^2^ = 8.205), whereas the *p* value obtained in the recessive model by Fisher exact test (TT vs. CT + CC) (*p* = 1.0000) and additive model (TT vs. CC) (*p* = 0.7122) were not significant. Following the co-dominant model, the CT genotype showed two folds higher risk of developing stress and depression (OR = 2.072, CI 1.28–3.35) compared to the CC genotype, while, the dominant model showed that the CT and TT genotype collectively imposed two times higher risk of stress compared to the CC genotype (OR = 2.011, CI 1.25–3.23). However, distribution of C and T alleles in cases and controls were significantly different (C allele 0.72 in cases vs 0.80 in control and T allele 0.28 in cases vs 0.20 in control) as depicted in Table 3 (*p* = 0.0159, χ^2^ = 5.807). The *SLC1A3* C3590T is not in Hardy-Weinberg equilibrium, indicating that the allele frequency may increase or decrease with time.

**Table 3:** Genotypic distribution of *SLC1A3* C3590T (rs 2,269,272) in relation with Stress and Depression

Genotype/Allele frequency	Cases (*N* = 108)	Controls (*N* = 205)	*p* (χ^2^)	Odds ratio (OR)	95% Confidence Interval (CI)
CC	50 (46.3%)	130 (63.4%)	0.0115 (8.934)*		
CT	55 (50.9%)	69 (33.7%)			
TT	3 (2.8%)	6 (2.9%)			
Linear by linear association (Armitage tests for trends)			0.010*		
Dominant Model (CT + TT vs. CC)			0.0052 (7.796)*	2.011	1.25–3.23
Co- dominant Model (CT vs. CC)			0.0042 (8.205)*	2.072	1.28–3.35
Recessive Model (TT vs. CT + CC)			1.0000	0.948	0.23–3.87
Additive Model (TT vs. CC)			0.7122	1.300	0.31–5.40
Allele frequency distribution					
Alleles	Cases	Controls	*p* (χ^2^)		
C	155 (0.72)	329 (0.80)	0.0159 (5.807)*		
T	61 (0.28)	81 (0.20)			

The table represents the genotypic frequency, the allelic frequency as well as odds - ratio for different models. Number of participants (*n*) = 313

* indicates *p* < 0.05

### SLC1A3 C869G (rs137852619) polymorphism analysis in stress and depression

**Fig. 2:**
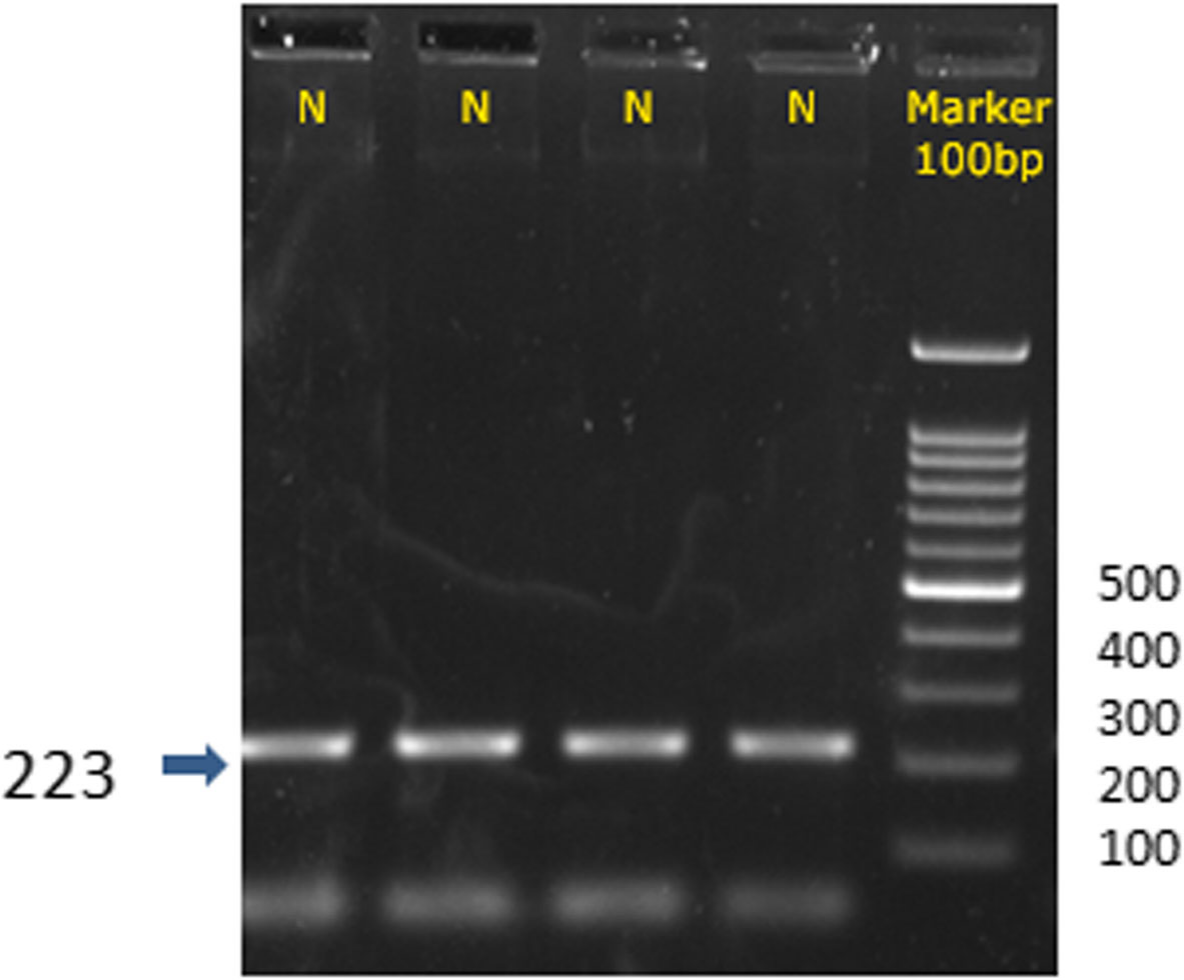
Restriction digestion by *Bst*U1 of *SLC1A3* C869G amplicon of representative individuals. This polymorphism was not found in the cohort of individuals selected and a full length amplicon of 223 bp, is seen in all the lanes. The cases and controls with reference to stress and depression showed no heterozygotes in control as well cases

In *SLC1A3* C869G, the amplicon normally has ‘G’ at 869th position which is not recognized by restriction endonuclease, *Bst*U1 and the full amplicon of 223 bp is expected after electrophoresis. But if ‘C’ is substituted in place of G, then a restriction site is created and 2 bands of 152 and 71 bp size are obtained; and the heterozygote shows three bands of 223, 152 and 71 bp. This polymorphism was not found in the present population under study so a full length amplicon of 223 bp was obtained (Fig. 2).

### Association of BDNF G196A (rs6265) polymorphism with stress and depression

**Fig. 3:**
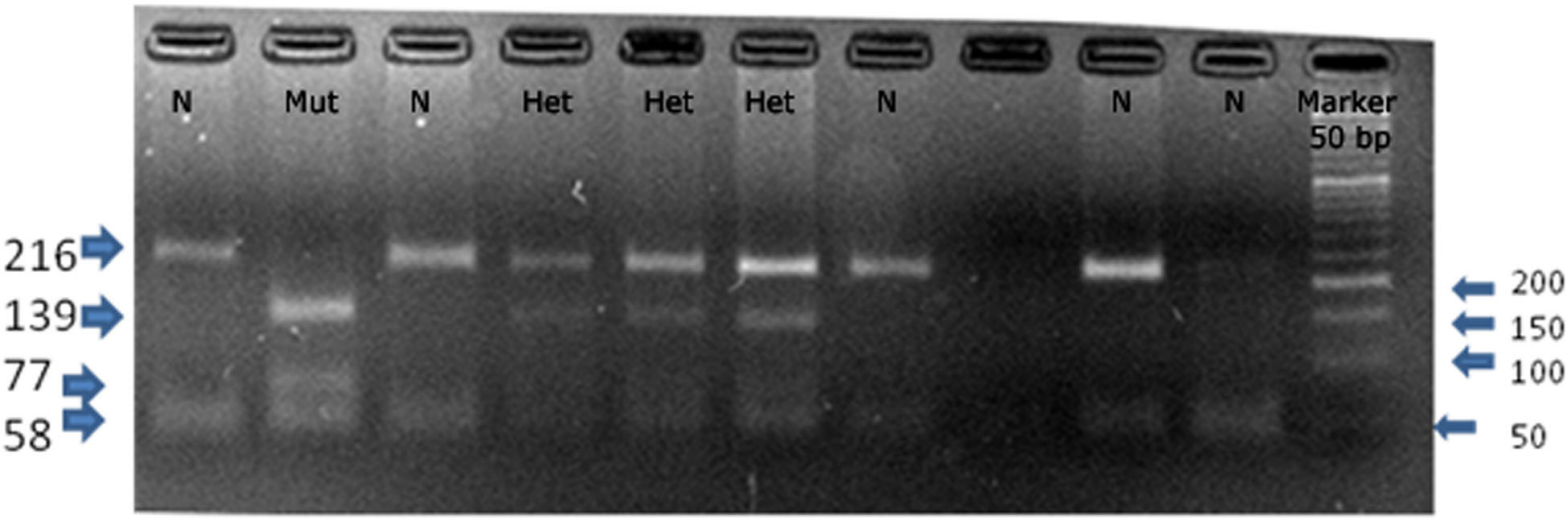
Restriction digestion of *BDNF* G196A amplicon by *Nla*III of representative individuals. The amplicon after digestion in normal condition (i.e. in presence of major allele G) N = major allele (homozygotes) which gives two bands of 216 and 58 bp (Lane 1, 3, 7, 9, 10); A substitution of A in place of G creates new restriction site, so Mut = minor allele (homozygotes) giving three bands of 139, 77 and 58 bp (lane 2) and Heterozygotes having both types of alleles forms 4 bands Het = heterozygotes, which give four bands of 216, 139, 77 and 58 bp (lane 4, 5, 6)

The genotypic distribution of *BDNF* G196A in both cases with stress and depression and normal controls is as shown in Table 4. *BDNF* G196A normally has G at 196th position which is not recognised by its restriction enzyme (*Nla* III) and cuts at 273rd position forming normal signal peptide and mature *BDNF* (two fragments amplicon 216 and 58 bp). But when there is substitution of A at 196 position, a new restriction site is created, therefore cut position is at 196th and 273rd positions producing 139, 77 and 58 bps fragments. Heterozygotes having both types of allele shows four fragments of 216, 139, 77 and 58 bps (Fig. 3). Hardy Weinberg equilibrium was calculated for *BDNF* G196A was found to be in equilibrium. The allelic frequencies showed uniformity in both cases and controls (G allele 0.80 in cases vs 0.79 in control and A allele 0.20 in cases vs 0.21 in control). Our data revealed lack of significant differences in GG, GA, AA: 64.8, 30.6 and 4.6%, respectively, for cases and 63.4, 31.2 and 5.4%, respectively, for controls. The distribution of the G and A alleles in cases and controls was not significantly different showing no association with stress and depression as depicted in Table 4 (*p* = 0.7534, χ^2^ = 0.0987).

**Table 4:** Genotypic distribution of *BDNF* G196A (rs6265) in relation with stress and depression (case) and unstressed (control)

Genotype/Allele frequency	Cases (*N* = 108)	Controls (*N* = 205)	*p* (χ^2^)
GG	70 (64.8%)	130 (63.4%)	0.9480 (0.1068)
GA	33 (30.6%)	64 (31.2%)	
AA	5 (4.6%)	11 (5.4%)	
Linear by linear association (Armitage tests for trends)			0.760
Allele frequency distribution			
Alleles	Cases	Controls	*p* (χ^2^)
G	173 (0.80)	324 (0.79)	0.7534 (0.0987)
A	43 (0.20)	86 (0.21)	

The table represents the genotypic frequency and the allelic frequency for different models. (*N* = 313)

## Discussion

The present study focuses on association of *SLC1A3* C3590T, C869G and *BDNF* G196A in case control studies with stress and depression in an eastern Indian population. Association with stress and depression was observed only in T allele of *SLC1A3* C3590T in a cohort from an eastern region of India (eastern Uttar Pradesh), suggesting that the minor allele (T) in *SLC1A3* C3590T may contribute to the risk of stress and depression together.

SLC1A3 gene in normal condition makes a protein called excitatory amino acid transporter 1 (EAAT1) expressed on mainly astrocyte membrane and involved in regulating excess glutamate concentration, which is found in synaptic spaces after synaptic transmission. EAAT1 is found throughout the brain, most abundantly in parts in connection to spinal cord (brain stem) and cerebellum (co-ordinates movements).

Since SLC1A3 forms a glial high-affinity glutamate transporter (GLAST) molecule regulating glutamate concentration after synaptic transmission, its anomalous expression may impair reuptake of glutamate, leading to prolonged synaptic activation and cytotoxicity in neurons and glial [40]. On the other hand as stated above, SLC1A3 is down-regulated in MDD (Major depressive disorder) patients, also reduced expression of *SLC1A3* gene has been observed in suicidal behaviour [41, 42]. In the present study, 3’UTR polymorphism (C3590T) is associated with stress as well as depression. Frequencies of CC genotype of *SLC1A3* C3590T were significantly higher in controls (63.4%) than in cases (46.3%). Calculations based on dominant model in Table 3 revealed that individuals with the CT and TT genotypes collectively showed more than two folds higher risk of developing stress and depression compared to those with CC genotype. Also, similar evidence is found from the co-dominant model, where CT genotype confers 2.072 fold higher risk of developing stress. Table 3 also shows that the frequency of C allele is much lower in cases (0.72) than in controls (0.80). The frequency of TT genotype and the minor allele frequency (MAF) of T allele (0.20) in our control group are also similar to those reported in previous studies with reference to depression and in other psychiatric disorders conducted in different populations [16, 43]. However, the frequencies of the CC genotype and C allele are lower in cases than in the control group and this is the first report of this polymorphism in Indian population. It can be hypothised that the 3’UTR polymorphism may decrease protein translation without change in protein conformation.

*SLC1A3* C869G, a missense SNP, is known to be pathogenic and mutation in this gene has been found to cause episodic ataxia type 6 [44]. This change from C to G, replacing amino acid proline to arginine at 290 position (P290R), impairs EAAT1 to remove glutamate from synaptic cleft. This impairment may overexcite certain neurons disrupting normal communication within neurons, which is visualised as uncoordinated movement in individuals with episodic ataxia. But the molecular mechanism in this is unknown, however in the present study *SLC1A3* C869G was not found to be associated with either stress or depression.

In a study by Bechtholt-Gompf [10] on rats it was shown that disruption of glutamate uptake from the synapse was linked to reduced sensitivity to reward, showing symptoms of depression. In another study on human autopsy samples, it was found that AMPA receptors (glutamate receptor) in anterior cingulated cortex were increased in major depressive disorder [45]. In an animal model study it was found that, mice knockout for AMPA subunit-1 showed higher helplessness, decrease in serotonin and nor-epinephrine levels and disturbed glutamate homeostasis. All these results establish that deregulated glutamate homeostasis leads to disruption of many functions and may lead to depression, too.

A support of our observation comes from a family based study by Turic et al. in 2005 conducted on Caucasian samples, that found significant association of T allele of *SLC1A3* (C3590T) with attention deficit hyperactivity disorder (ADHD) [43]. The association of the same polymorphism was also later shown by Murphy et al. [16] in suicidal victims where SLC1A3 expression was shown to be highly reduced [15]. In the present study the ‘T’ allele was associated with stress and depression, whereas the C allele (major allele) acts as a protective allele. Therefore, it could be speculated that like in suicidal victims, in the above study also due to presence of T allele, the expression of the gene was reduced predisposing the individuals for stress and/or depression. On the other hand a study done on samples from South Korea on mood disorder by Choi et al. [46], did not find association of suicidal behaviour with this polymorphism.

*BDNF* is the most studied gene, which is shown to be associated with many different mental disorders and is known to interact with various developmental pathways [47]. The trafficking and localization of BDNF appears to be controlled by its pro-domain. A SNP in the pro-domain of BDNF, converts the 66th amino acid valine into methionine (Val66Met) which affects dendritic trafficking and synaptic localization of BDNF as well as impairs its secretion. Human subjects carrying the Val66Met SNP exhibit deficits in short-term episodic memory and show abnormal hippocampal activation.

*BDNF* G196A polymorphism reduces the expression of the gene and activates apoptotic pathway [48, 49] and in other populations as US Caucasians, American Indian, African American, Chinese a significant association of this polymorphism was shown with depression cases [50–52] and several psychiatric disorders such as anxiety, obsessive compulsive disorder, depression, schizophrenia, psychosis and eating disorder [36, 53]. *BDNF* G196A, “A” (minor allele) has been considered as the risk factor because this minor allele carriers have a reduced activity dependent secretion of BDNF [29, 54]. However G allele (major allele) has been reported to be associated with bipolar disorder [55, 56] and substance use [57, 58] with increased BDNF activity [59–62]. Also, in Mexican- American population GG genotype (Val homozygote) has been found to be associated with increased chance of depression [63]. A meta-analysis on different ethnic groups performed by Li et al. [31] found significant association of G allele with bipolar in Europeans but not in Asians. And allele A carriers show an increased propensity towards Parkinson’s disease in Europeans but not in Asians. However, a positive association was found with suicidal behaviour in Asians, Caucasians but not in Europeans. But contrary to this, in the present study no association of G196A was found with stress and depression. In the present study, interestingly the frequencies of AA (minor allele of *BDNF* G196A) was more in controls than in cases, however the χ^2^ = 0.0987 was not significant, although, it is known that *BDNF* G196A allele A have a variable frequency ranging between 0% in Africa upto72% in Asia [30]. This allelic frequency distribution in eastern Indian population may be of significance and needs a wider study. Further GWAS studies in recent years have identified several genetic loci but did not find any association of *BDNF* G196A with stress and depression [29].

## Conclusion

We conclude that *SLC1A3* C3590T (rs2269272) polymorphism is associated with stress and depression in eastern Indian population studied (*p* = 0.0042, OR = 2.072). This allele increases the risk by two folds for stress and depression in the present population, therefore presence of this allele in an individual may predict the development of stress and depression in future. This knowledge may also help in development of drug for those having this allele and may also help in the understanding of pathophysiology of the disease and identification of better drug.

## Data Availability

The data sets analysed throughout the present study are available in the NCBI database (https://www.ncbi.nlm.nih.gov/). The dataset(s) supporting the conclusions of this article is included within the article.

## Abbreviations

BDNF: Brain derived neurotrophic factor
SLC1A3: Solute Carrier Family 1 Member 3 (sodium dependent, high affinity amino acid transporter)
PSS: Percieved stress scale
CES-D: Center for epidemiologic Studies Depression
PCR: Polymerase Chain Reaction
SNP: Single Nucleotide polymorphism
SLC1: Solute Carrier Family 1 (glutamate/neutral aminoacid transporter family)
EAAT: Excitatory amino acid transporters
AMPA: α-amino-3-hydroxy-5-methyl-4-isoxazole propionic acid (glutamate receptor)
NMDA: N-methyl-D-aspartate receptor (inotropic glutamate receptor)
GWAS: Genome-Wide Association Studies
tSNARE1: t-SNARE domain containing 1
SLC1A2: Solute Carrier Family 1 Member 2 (glial glutamate transporter)
SLC17A7: Solute Carrier Family 17 Member 7 (sodium dependent inorganic phosphate co-transporter)
UTR: Untranslated region
Val66Met: Valine to methionine at codon 66
Met: Methionine
Val: Valine
DSM-5: Diagnostic and Statistical Manual of Mental Disorders, 5th edition
°C: Degree Celcius
μl: microliter
rpm: revolutions per minute
min: minute
sec: second
SDS: Sodium dodecyl sulfate
DNA: Deoxyribonucleic acid
EDTA: Ethylene diamine tetra acetic acid
BLAST: Basic Local Alignment Search Tool
SPSS: Statistical Package for the Social Sciences
CI: Confidence interval
OR: Odds ratio
bp: base pairs
GLAST: Glial high-affinity glutamate transporter
MDD: Major depressive disorder
MAF: Minor allele frequency
ADHD: Attention deficit hyperactivity disorder
